# Correlation Between Electroretinogram and Visual Prognosis in Metallic Intraocular Foreign Body Injury

**DOI:** 10.3389/fmed.2021.688305

**Published:** 2021-06-24

**Authors:** Xiaoting Mai, Fangyi Ling, Yuting Gong, Jialin Chen, Hongjie Lin, Haoyu Chen

**Affiliations:** Joint Shantou International Eye Center, Shantou University and the Chinese University of Hong Kong, Shantou, China

**Keywords:** intraocular foreign body, electroretingraphy, visual prognosis, eye injuries, penetrating injuries to eye

## Abstract

**Purpose:** This study aims to investigate the correlation between electroretinogram (ERG) and visual outcome in eyes with metallic intraocular foreign body (IOFB) injury.

**Methods:** Cases with metallic IOFB injuries with preoperative ERG from January 2008 to May 2020 were reviewed retrospectively. Five ERG responses were recorded, including rod response, maximal response, oscillatory potentials, cone response, and 30-Hz flicker. The results were compared between the affected and the contralateral eyes. All patients received surgery to remove IOFBs. The correlation between amplitudes, implicit times, and grades of ERG with final best-corrected visual acuity (BCVA) was analyzed.

**Results:** A total of 33 eyes of 33 patients were included. The eyes with IOFB had generally delayed implicit time and reduced amplitude in all waves. The maximum change was found in oscillatory potentials S3 and N1 (0.42 ± 0.42 and 1.95 ± 1.97 of the fellow eyes, respectively, *p* < 0.05). All amplitudes were negatively correlated with the final BCVA (rs: −0.676 to −0.459, all *p* < 0.05). In contrast, all implicit times were positively correlated with final BCVA, although, some of them were not statistically significant (rs: 0.035 to 0.687). Among them, oscillatory potential P3 has the highest correlation coefficient (rs = 0.687, *p* < 0.001). All grades of ERG waves were statistically correlated with the final BCVA (rs: −0.596 to −0.664, all *p* < 0.001).

**Conclusions:** ERG can be used to assess visual outcome in metallic IOFB injury after surgery. Oscillatory potentials provided the most significant responses.

## Introduction

Intraocular foreign body (IOFB) injury is a specific type of open globe injury, which results in mechanical impact and metallic toxicity to intraocular tissue (1–3). Retained metallic IOFB can cause siderosis bulbi. Anterior segment examination may reveal iron deposits on the cornea and anterior capsule, iris heterochromia, pupillary mydriasis, cataract, and glaucoma. Retina toxicity usually manifests as retinal arteriolar narrowing and sheathing and pigmentary retinal degeneration (4). Our previous study found that photoreceptor damage and inner retinal ischemia are two major findings of metallic toxicity to the retina on OCT.

Retinal electrophysiological examination in metallic IOFB injury has been shown to be associated with delayed implicit time and reduced amplitude in both a wave and b wave, suggesting both inner and outer retinal impairment (5–8). Electroretinogram (ERG) response can also be used to monitor retinal toxicity in IOFB and provide a reference for surgical intervention (9). It was also reported that visual acuity and ERG response improved after the surgical removal of IOFB (6). However, the visual outcome was highly dispersive, and some complications such as retinal detachment and endophthalmitis predicted poor visual outcomes (10). It remains unknown whether the retinal toxicity quantified by the ERG responses can be used to predict the visual outcome of IOFB. Furthermore, which component of the ERG responses is the most significant change also needs further investigation.

The purpose of our study was to quantify and grade the ERG responses after a metallic IOFB injury and analyze the correlation between ERG and visual outcome so as to facilitate visual prognosis after a metallic IOFB injury.

## Methods

This retrospective study reviewed all metallic IOFB injury cases between January 2008 and May 2020 in Joint Shantou International Eye Center (JSIEC) of Shantou University and the Chinese University of Hong Kong. This study was approved by the JSIEC Institutional Review Board. Informed consent was waived because of the retrospective nature of this study.

The inclusion criteria were as follows: (1) diagnosis of metallic IOFB injury and (2) ERG was performed after IOFB injury and before its removal. The exclusion criteria were as follows: (1) lack of preoperative ERG data of the injured eye, (2) ERG was conducted using other models rather than Retiport32, (3) presence of retinal detachment or macular hole, and (4) history of retina disease.

Data on history, best-corrected visual acuity (BCVA), duration of foreign body, ERG, and follow-up time were collected. Visual acuity was measured using the international standard logarithmic visual acuity chart and converted to minimum resolution angular logarithm (logMAR) unit for statistical analysis. Finger counting was converted to 2.0 logMAR, hand motion was converted to 2.3 logMAR, and light perception was converted to 3.0 logMAR (11).

The pupils were dilated with 0.5% tropicamide and 0.5% phenylephrine, and the other eye was occluded. Standardized full-field ERGs were elicited with Ganzfeld stimuli using the commercial ERG system (Retiport32; Roland Consult, German). Five responses were recorded, including (1) dark-adapted 0.01 ERG (rod response), (2) dark-adapted 3.0 ERG (maximal response), (3) dark-adapted 3.0 oscillatory potentials (oscillatory potentials, OPs), (4) light-adapted 3.0 ERG (cone response), and (5) light-adapted 3.0 flicker ERG (30-Hz flicker) (12). The implicit period and the amplitude were recorded. For non-recordable responses, the amplitude was set as 0, while the implicit time was set as a missing value. The responses were graded into five levels as follows: non-recordable, severely subnormal (5–40% of normal values), moderately subnormal (40–70% of normal values), mildly subnormal (70–90% of normal values), and normal (90–110% of normal values).

The ratios of the parameters of the affected eye to the contralateral eye was calculated. Paired *t*-test was used to compare the ERG parameters between the injured and fellow eyes. Wilcoxon ranked test was used to compare the preoperative BCVA with the final BCVA. Spearman's correlation was used to analyze the relationship between the ERG implicit period, amplitude, and grade with the final BCVA. Statistical analyses were performed using SPSS Statistics software (version 21.0, SPSS Inc., Chicago, IL). Statistical significance was considered when *p* < 0.05.

## Results

**Figure 1:**
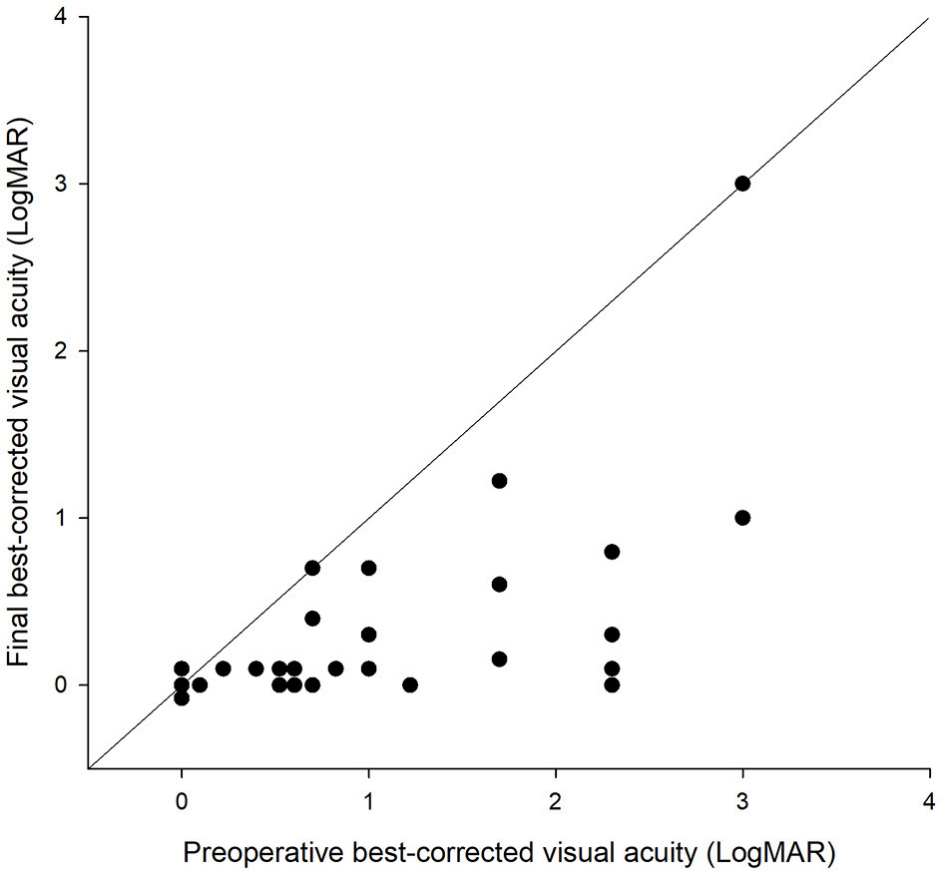
Scatter plot of the preoperative and final best-corrected visual acuity (BCVA) in each intraocular foreign body-injured eye. The dots on the oblique line indicate no change of BCVA after the operation. The dots below the oblique line indicate an improvement of BCVA. The dots above the oblique line indicate a worsening of BCVA after the operation.

A total of 33 eyes of 33 patients were included. There were 30 males and three females (10 right eyes, 23 left eyes). The median IOFB duration time was 291 days. The median follow-up time was 126 days. The location of the wound, length of the wound, lens, location of IOFB, character of IOFB, and type of surgery of each patient are listed in Table 1. There was no patient with direct macular injury. There was no significant correlation between the IOFB duration time with preoperative BCVA or final BCVA (*r* = 0.241 and 0.270, respectively; both *p* > 0.05). BCVA improved in 29 eyes, remained unchanged in three eyes, and decreased in one eye after surgery (Figure 1). The mean BCVA improved from 1.23 ± 0.92 logMAR preoperatively to 0.36 ± 0.55 logMAR postoperatively (*p* < 0.001).

**Table 1:** Demographic and clinical information of the included subjects.

**Gender**	**Age**	**Eye**	**Injury-ERG (d)**	**ERG-IOFBR (d)**	**Wound location**	**Wound length**	**Lens**	**Location of IOFB**	**Magnetic**	**Cataract surgery**	**IOFB removal**
Female	41	OD	724	6	Sclera	Unknown	Cataract	Intravitreal	Non-magnetic	Y	PPV
Male	41	OS	33	0	Peripheral cornea	5 mm	Cataract	Peripheral retina	Magnetic	N	External
Male	48	od	3,643	8	Sclera	1 mm	Cataract	Peripheral retina	Magnetic	Y	PPV
Male	22	OS	3	3	Peripheral cornea	3 mm	Cataract	Intravitreal	Magnetic	Y	PPV
Male	31	OS	2,488	76	Sclera	1 mm	Cataract	Intravitreal	Magnetic	Y	PPV
Male	34	OS	332	34	Peripheral cornea	4 mm	Pseudophakia	Pars plana	Magnetic	N	PPV
Male	37	OS	716	15	Sclera	1.5 mm	Cataract	Pars plana	Magnetic	Y	PPV
Male	40	OS	16	0	Sclera	Unknown	Cataract	Intravitreal	Magnetic	Y	PPV
Male	45	OS	3	1	Peripheral cornea	1 mm	Clear	Intracameral	Magnetic	N	AC
Male	21	OS	17	1	Paracentral cornea	6 mm	Cataract	Peripheral retina	Magnetic	Y	PPV
Male	23	OS	258	33	Paracentral cornea	10 mm	Cataract	Peripheral retina	Non-magnetic	Y	PPV
Male	35	OS	4	1	Peripheral cornea	2 mm	Local cataract	Peripheral retina	Magnetic	Y	PPV
Male	16	OS	2	2	Peripheral cornea	3 mm	Local cataract	Peripheral retina	Magnetic	N	PPV
Male	22	OD	1	0	Sclera	1 mm	Clear	Intravitreal	Magnetic	N	PPV
Male	20	OS	91	0	Sclera	1 mm	Clear	Vascular arcade	Magnetic	N	PPV
Male	41	OS	674	786	Peripheral cornea	2 mm	Local cataract	Peripheral retina	Magnetic	N	PPV
Female	43	OS	54	7	Peripheral cornea	1 mm	Cataract	Intralenticular	Magnetic	Y	AC
Male	31	OD	25	1	Peripheral cornea	2 mm	Local cataract	Intravitreal	Magnetic	N	External
Male	60	OS	3,650	1	Peripheral cornea	2 mm	Mild cataract	Intracameral	Magnetic	N	AC
Male	61	OS	2,190	4	Peripheral cornea	1 mm	Cataract	Peripheral retina	Magnetic	Y	PPV
Male	46	OS	723	8	Sclera	1 mm	Cataract	Peripheral retina	Magnetic	Y	PPV
Female	42	OD	30	0	Limbus	1 mm	Cataract	Intravitreal	Magnetic	Y	PPV
Male	44	OD	3,651	3	Sclera	Unknown	Cataract	Peripheral retina	Non-magnetic	Y	PPV
Male	58	OS	3,648	4	Peripheral cornea	2 mm	Local cataract	Intracameral	Non-magnetic	N	AC
Male	35	OS	3,637	14	Peripheral cornea	1 mm	Cataract	Peripheral retina	Magnetic	Y	PPV
Male	30	OS	245	0	Peripheral cornea	1 mm	Cataract	Intravitreal	Magnetic	Y	PPV
Male	28	OD	730	1	Sclera	1 mm	Cataract	Peripheral retina	Magnetic	Y	PPV
Male	24	OS	60	1	Sclera	Unknown	Cataract	Peripheral retina	Magnetic	Y	PPV
Male	41	OD	2,555	1	Sclera	1 mm	Cataract	Peripheral retina	Magnetic	Y	PPV
Male	56	OD	401	1	Sclera	4 mm	Pseudophakia	Pars plana	Magnetic	N	External
Male	43	OD	1,460	1	Sclera	Unknown	Local cataract	Peripheral retina	Magnetic	Y	PPV
Male	33	OS	31	1	Paracentral	3 mm	Local cataract	Peripheral retina	Magnetic	Y	PPV
Male	26	OS	21	1	Sclera	1 mm	Clear	Intravitreal	Magnetic	N	PPV

*d, days; ERG, electroretinography; IOFB, intraocular foreign body; IOFBR, intraocular foreign body removal; PPV, pars plana vitrectomy; AC, anterior chamber*.

The implicit times were delayed, and the amplitudes were reduced in all ERG responses (Table 2). The ratios of implicit time in the injured eye compared to the contralateral eye ranged from 1.12 to 1.95 (all *p* < 0.05), and the ratios of amplitude ranged from 0.42 to 0.66 (all *p* < 0.05, except the Ops4, whose *p*-value was 0.060). The maximum ratios were OS3 (0.42 ± 0.42 of the fellow eye) and OP N1 (1.95 ± 1.97 of the fellow eye).

**Table 2:** Results of preoperative electroretinogram (ERG) response parameters and their correlation with the final best-corrected visual acuity.

**Response**	**Parameter**	***N***	**Mean ± SD**	**Ratio to the** **fellow eye**	**Paired *t*-test**	**Spearman correlation with baseline BCVA**		**Spearman correlation with final BCVA**		**Spearman correlation with injury to ERG time**	
					***P*-value**	**Coefficient**	***P*-value**	**Coefficient**	***P*-value**	**Coefficient**	***P*-value**
Rod response	b (ms)	23	103.13 ± 12.86	1.45 ± 0.53	<0.001	0.422	0.045	0.151	0.493	0.244	0.261
	b-wave (μV)	33	138.80 ± 126.00	0.46 ± 0.41	<0.001	−0.438	0.011	−0.607	<0.001	−0.256	0.151
Maximal response	a (ms)	30	24.63 ± 2.30	1.22 ± 0.33	0.001	0.508	0.004	0.565	0.001	0.589	0.001
	b (ms)	30	51.53 ± 7.19	1.12 ± 0.31	0.037	0.394	0.031	0.106	0.576	0.131	0.489
	a-wave (μV)	33	163.30 ± 112.83	0.56 ± 0.36	<0.001	−0.468	0.006	−0.459	0.007	−0.204	0.256
	b-wave (μV)	33	323.15 ± 228.53	0.64 ± 0.65	<0.001	−0.405	0.019	−0.507	0.003	−0.163	0.364
Oscillatory potentials	N1 (ms)	28	16.64 ± 1.91	1.95 ± 1.97	0.014	0.326	0.090	0.446	0.017	0.190	0.334
	P1 (ms)	28	20.82 ± 1.91	1.76 ± 1.78	0.010	0.471	0.011	0.629	<0.001	0.345	0.072
	N2 (ms)	28	23.82 ± 2.75	1.64 ± 1.66	0.008	0.450	0.016	0.445	0.018	0.394	0.038
	P2 (ms)	28	29.07 ± 3.93	1.57 ± 1.58	0.004	0.469	0.012	0.512	0.005	0.410	0.030
	N3 (ms)	27	32.22 ± 4.30	1.54 ± 0.903	0.003	0.487	0.010	0.54	0.004	0.418	0.030
	P3 (ms)	27	35.07 ± 3.81	1.49 ± 0.82	0.002	0.507	0.007	0.687	<0.001	0.526	0.005
	N4 (ms)	25	38.84 ± 4.41	1.48 ± 0.73	0.001	0.422	0.035	0.318	0.121	0.501	0.011
	P4 (ms)	25	41.92 ± 4.38	1.42 ± 0.65	0.002	0.357	0.080	0.288	0.163	0.591	0.002
	OS1 (μV)	33	18.96 ± 14.60	0.48 ± 0.32	<0.001	−0.462	0.007	−0.626	<0.001	−0.358	0.041
	OS2 (μV)	33	53.22 ± 42.07	0.50 ± 0.39	<0.001	−0.490	0.004	−0.611	<0.001	−0.267	0.133
	OS3 (μV)	33	17.82 ± 18.64	0.42 ± 0.42	<0.001	−0.427	0.013	−0.676	<0.001	−0.278	0.117
	OS4 (μV)	33	11.32 ± 13.73	0.66 ± 0.96	0.060	−0.236	0.186	−0.499	0.003	−0.074	0.680
	OS1 + OS2 + OS3 (μV)	33	89.99 ± 70.57	0.47 ± 0.35	<0.001	−0.472	0.006	−0.644	<0.001	−0.302	0.087
Cone response	a (ms)	28	17.04 ± 1.73	1.29 ± 0.53	0.005	0.417	0.027	0.439	0.020	0.456	0.015
	b (ms)	28	33.50 ± 2.94	1.25 ± 0.43	0.003	0.496	0.007	0.523	0.004	0.343	0.074
	a-wave (μV)	33	33.57 ± 22.17	0.59 ± 0.38	<0.001	−0.481	0.005	−0.595	<0.001	−0.275	0.122
	b-wave (μV)	28	96.62 ± 76.29	0.54 ± 0.40	<0.001	−0.473	0.005	−0.602	<0.001	−0.289	0.103
30-Hz flicker	N1 (ms)	29	15.31 ± 2.69	1.36 ± 0.62	0.003	0.532	0.003	0.413	0.026	−0.008	0.967
	P1 (ms)	29	31.79 ± 3.52	1.31 ± 0.43	<0.001	0.462	0.012	0.500	0.006	0.199	0.301
	N1-P1 (μV)	33	70.63 ± 54.27	0.54 ± 0.40	<0.001	−0.371	0.033	−0.539	0.001	−0.191	0.287
	30-Hz amplitude (μV)	33	32.44 ± 22.19	0.56 ± 0.38	<0.001	−0.476	0.005	−0.648	<0.001	−0.275	0.122

*ms, implicit period in millisecond; μV, amplitude in microvolt; BCVA, best-corrected visual acuity*.

**Figure 2:**
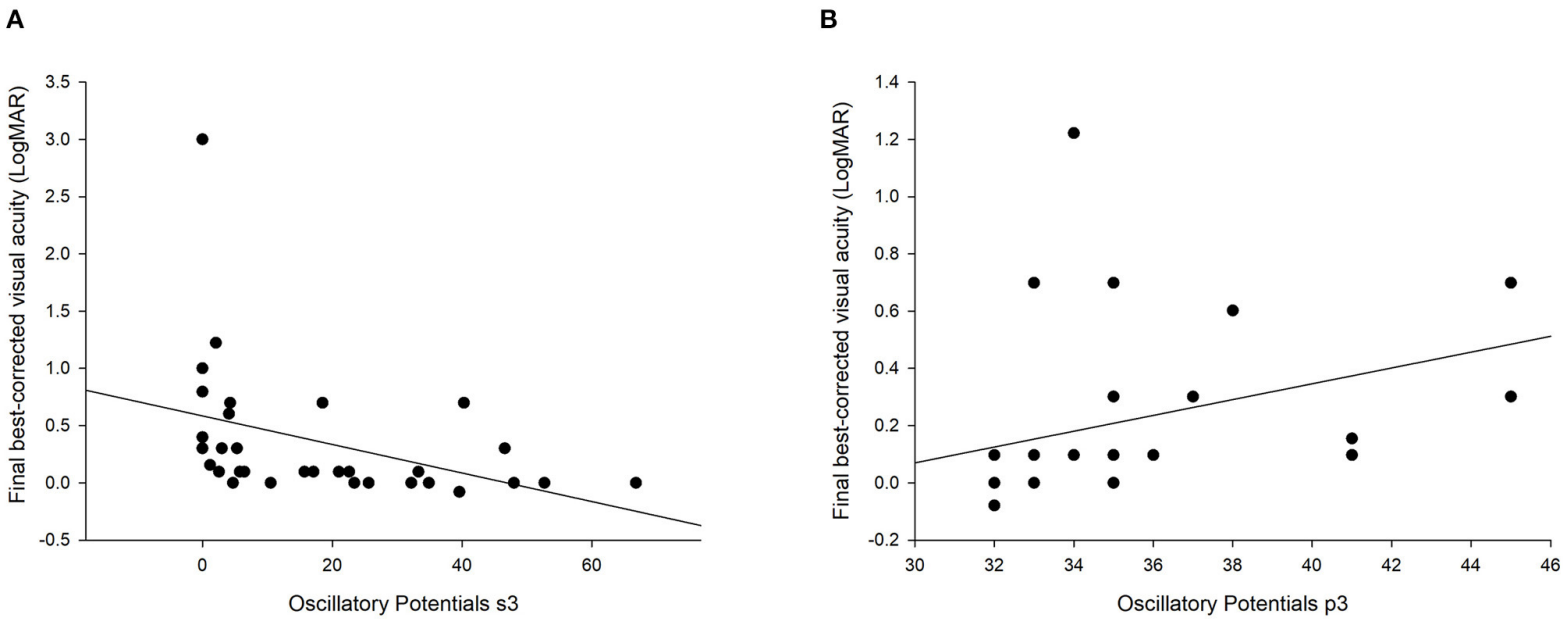
Scatter plots of the oscillatory potentials OS3 **(A)** and P3 **(B)** with the final best-corrected visual acuity. The lines represent the regression lines.

**Figure 3:**
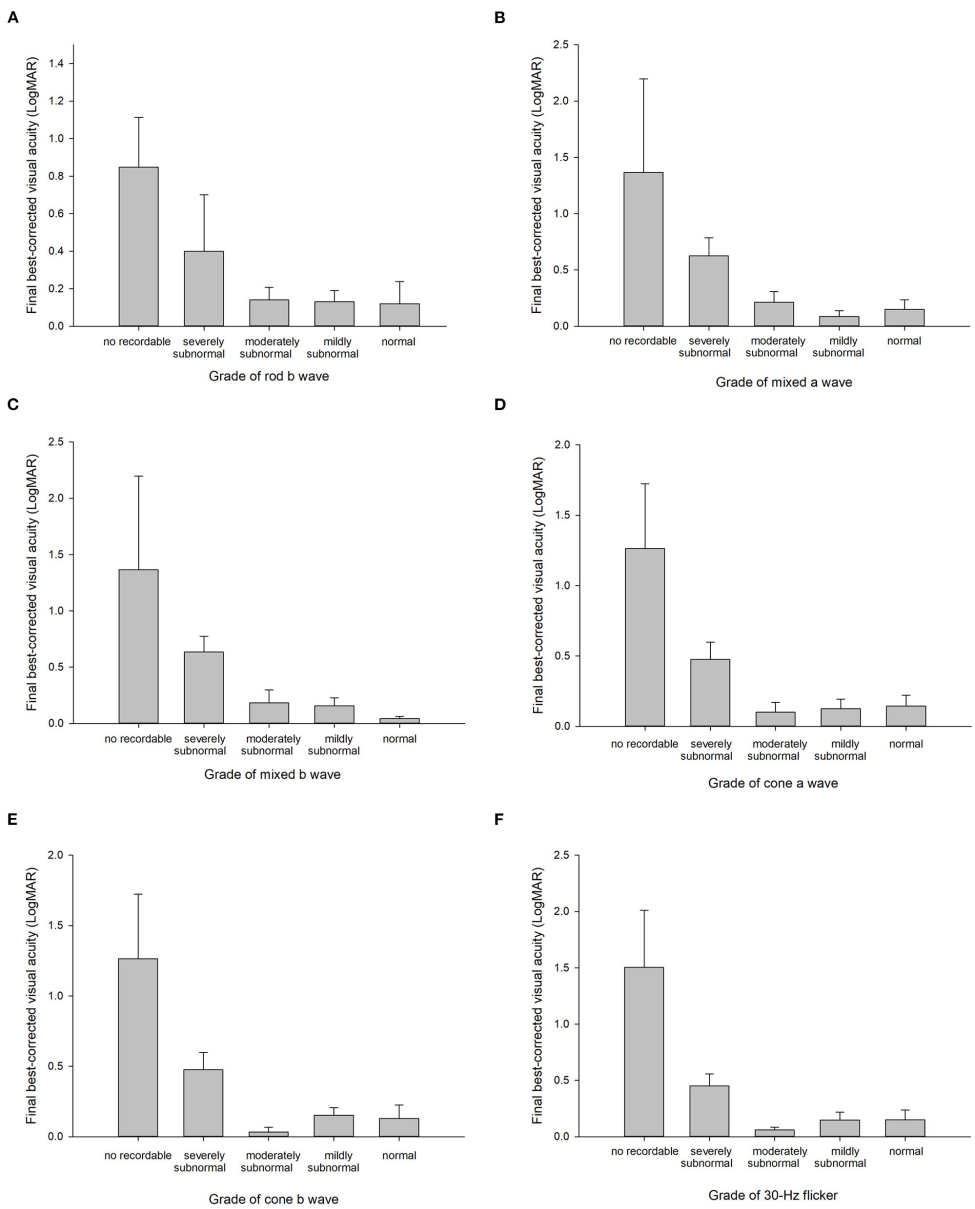
Final best-corrected visual acuity in the different grades of rod b wave **(A)**, mixed a wave **(B)**, mixed b wave **(C)**, cone a wave **(D)**, cone b wave **(E)**, and 30-Hz flicker **(F)**. The error bars represent standard errors.

All amplitudes were negatively correlated with the final BCVA (rs: −0.676 to −0.459, all *p* < 0.05, Table 2). The maximum correlation was with OS3 (Figure 2A). In contrast, the implicit times were positively correlated with the final BCVA, although, some of them were not statistically significant (rs: 0.072 to 0.687, Table 2). The maximum correlation was OP P3 (Figure 2B). The gradings of all the six ERG waves were statistically correlated with the final BCVA, with the correlation coefficient ranging from −0.596 to −0.664 (all *p* < 0.001, Figure 3). The IOFB duration time was positively correlated with the implicit time of Max a wave, OP N2, P2, N3, P3, N4, P4, and cone a wave and negatively correlated with amplitude of OP OS1 (Table 2).

**Figure 4:**
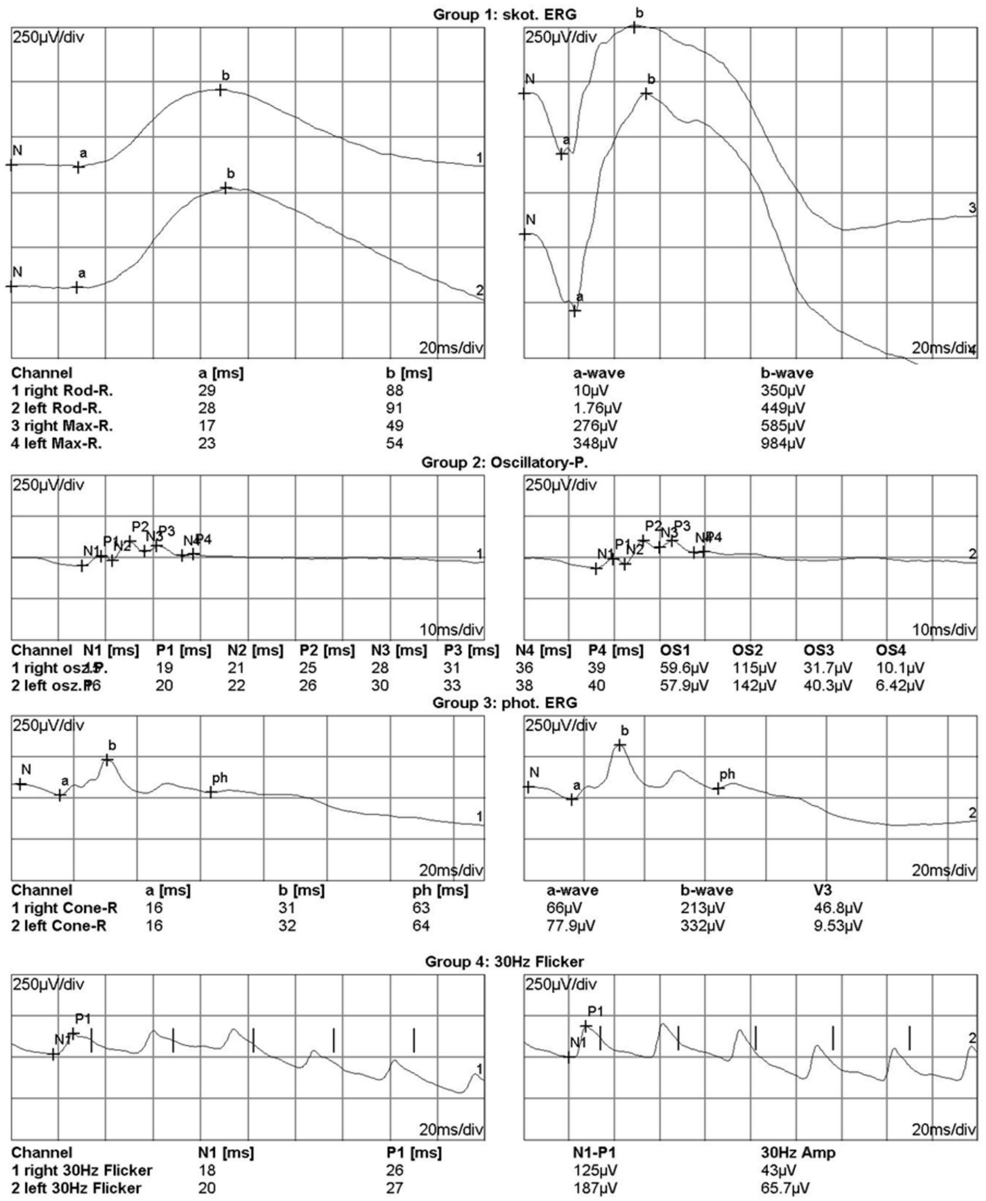
Electroretinogram of a case with enhanced electroretinography responses in the left eye which had intraocular foreign body injury at 1 day before. In the right panels were the responses of the right eye and in the left panels were the responses of the left eye. The iron intraocular foreign body passed through the cornea and finally located in the peripheral retina. The initial best-corrected visual acuity (BCVA) was 0 and 1.0 logMAR for the right and left eye, respectively. The final BCVA was 0 and 0.7 logMAR for the right and left eye, respectively.

There were two eyes with completely non-recordable responses in all waves. In the first case, both the baseline and final BCVA were light perception, while in the second case, the BCVA improved from 1.2 logMAR preoperatively to 0.8 LogMAR at the last follow-up. There was a case with a supernormal ERG response. The time from IOFB injury to ERG examination was 1 day. The iron IOFB located at the peripheral retina and the preoperative ERG showed a slight increase in the amplitudes of all ERG waves in the injured eye (Figure 4). There was a corneal scar at the paracentral cornea. The initial and final BCVA was 1.0 and 0.7 logMAR, respectively.

## Discussion

In this study, we reviewed the data of 33 eyes with metallic IOFB and found a reduction in the ERG wave amplitudes and delay in implicit periods. The maximum changes were at the OP OS3 and N1. All the amplitudes were negatively correlated with the final BCVA. In contrast, all implicit times were positively correlated with the final BCVA, although, some were not statistically significant. Among them, OP OS3 and P3 had the highest correlation coefficient. The gradings of all the six ERG waves were also statistically correlated with the final BCVA.

Our results showed that metallic IOFB leads to a decrease in amplitudes and implicit periods, which is similar to the literature results (5). There were several components in the five ERG responses. Different components represent the function of different cells. The general reduction in ERG responses suggests that all the retinal cells, including rod and cone photoreceptors, bipolar cells, and vascular function are affected in IOFBs.

A previous study investigated the prognostic value of ERG in severe recent ocular trauma (13). However, there were only seven eyes with IOFBs, and no quantitative data was analyzed. Our results showed that most of the waves differed between the affected and the contralateral eyes and correlated with the final BCVA. OPs were the most significant components. OPs record the activity of inhibitory synaptic circuits within the inner plexiform layer, representing the function of retinal microcirculation (12). It has been reported that b wave and OPs were enhanced, and visual outcomes were good in three eyes with early stage of IOFB injury (14). In another study, the amplitudes of rod and cone ERGs and the OPs were reduced after the injury. After the surgery, the amplitudes of rods and cones were markedly improved, but the OP amplitudes remained unchanged (6). These results suggested that the maintenance of retinal microcirculation after the injury is helpful to the recovery of postoperative vision.

ERG can detect retinal toxicity in metallic IOFBs. It has been reported that retinal dysfunction caused by retinal toxicity of metallic IOFB is reversible in the early stages (6), but the reversal was partial (15). Although, our study did not investigate ERG changes after the surgical removal of IOFB, our results showed that most cases achieve visual improvement. The preoperative ERG results were correlated with visual outcomes. It suggests that retinal damage may be at least partially irreversible in IOFB patients, especially in patients with non-recordable or severely subnormal responses. Our results provide a reference for predicting the visual prognosis in IOFB.

In the two patients with non-recordable ERG in all responses, the final BCVA was light perception in one patient but 0.8 logMAR in the other. It was also reported in the literature that non-recordable ERG might not indicate a poor visual outcome (16). Therefore, surgical removal is still recommended for these patients.

The median time of IOFB retainment was 291 days in this case series. It suggests that this group of patients has delayed presentation to ophthalmologists. There are several reasons of delay in visiting a physician. In some patients, the wound was small and the initial visual acuity was good. Some patients lack medical insurance and have a financial problem, and there was selection bias in that only patients whose wound was closed and IOFB was retained received ERG examination.

We also had a patient with enhanced ERG responses. Similar results were also reported in a case report (17). The enhanced responses occur in the early stage, which may be due to metal ions increasing the intraocular fluid's electrical conductivity and thus changing the resting potential of retinal cells (8). Our case had a history of IOFB injury at 1 day before the ERG examination. The patient's final BCVA (logMAR 0.7) may be due to the corneal scar rather than the retinal toxicity.

Our study has the advantage of a relatively large sample size which allows us to analyze the correlation between ERG components and visual prognosis, and we investigated all the components in five ERG responses and identified the most significant components. Our finding would help physicians to select the appropriate ERG parameter and predict the visual outcome of IOFB patients.

We recognize some limitations in the current study. Firstly, it is a retrospective study, and there may be a selection bias. Secondly, the visual outcome in IOFB patients may be affected by several factors, such as corneal scar. Thirdly, we did not conduct an ERG examination after IOFB removal and did not have longitudinal data.

In conclusion, in our study, ERG response was correlated with visual outcomes after a metallic IOFB injury. Oscillatory potentials were the most important quantitative parameters.

## Data Availability Statement

The raw data supporting the conclusions of this article will be made available by the authors, without undue reservation.

## Ethics Statement

The studies involving human participants were reviewed and approved by Institutional Review Board of Joint Shantou International Eye Center. Written informed consent for participation was not required for this study in accordance with the national legislation and the institutional requirements.

## Author Contributions

XM contributed to data collection and first draft. FL contributed to data analysis. YG contributed to data collection. JC and HL contributed to ERG examination. HC contributed to study design and manuscript revision. All authors contributed to the article and approved the submitted version.

## Conflict of Interest

The authors declare that the research was conducted in the absence of any commercial or financial relationships that could be construed as a potential conflict of interest.
